# Ketamine treatment for individuals with treatment-resistant depression: longitudinal qualitative interview study of patient experiences

**DOI:** 10.1192/bjo.2020.132

**Published:** 2020-12-07

**Authors:** Karen Lascelles, Lisa Marzano, Fiona Brand, Hayley Trueman, Rupert McShane, Keith Hawton

**Affiliations:** Oxford Health NHS Foundation Trust; and Centre for Suicide Research, Department of Psychiatry, University of Oxford, UK; Faculty of Science and Technology, Middlesex University, UK; Oxford Health NHS Foundation Trust; and Centre for Suicide Research, Department of Psychiatry, University of Oxford, UK; Oxford Health NHS Foundation Trust, UK; Department of Psychiatry, University of Oxford; and Oxford Health NHS Foundation Trust, UK; Centre for Suicide Research, Department of Psychiatry, University of Oxford; and Oxford Health NHS Foundation Trust, UK

**Keywords:** ketamine, treatment-resistant depression, mood, suicidal ideation, side-effects

## Abstract

**Background:**

Ketamine has recently received considerable attention regarding its antidepressant and anti-suicidal effects. Trials have generally focused on short-term effects of single intravenous infusions. Research on patient experiences is lacking.

**Aims:**

To investigate the experiences over time of individuals receiving ketamine treatment in a routine clinic, including impacts on mood and suicidality.

**Method:**

Twelve fee-paying patients with treatment-resistant depression (6 females, 6 males, age 21–70 years; 11 reporting suicidality and 6 reporting self-harm) who were assessed as eligible for ketamine treatment participated in up to three semi-structured interviews: before treatment started, a few weeks into treatment and ≥2 months later. Data were analysed thematically.

**Results:**

Most participants hoped that ketamine would provide respite from their depression. Nearly all experienced improvement in mood following initial treatments, ranging from negligible to dramatic, and eight reported a reduction in suicidality. Improvements were transitory for most participants, although two experienced sustained consistent benefit and two had sustained but limited improvement. Some participants described hopelessness when treatment stopped working, paralleled by increased suicidal ideation for three participants. The transient nature and cost of treatment were problematic. Eleven participants experienced side-effects, which were significant for two participants. Suggestions for improving treatment included closer monitoring and adjunctive psychological therapy.

**Conclusions:**

Ketamine treatment was generally experienced as effective in improving mood and reducing suicidal ideation in the short term, but the lack of longer-term benefit was challenging for participants, as was treatment cost. Informed consent procedures should refer to the possibilities of relapse and associated increased hopelessness and suicidality.

## Ketamine for treatment of depression

The use of ketamine in the treatment of depression has attracted much attention, with some viewing it as the first ‘breakthrough’ new treatment for affective disorders in the past few decades.^1^ Ketamine has primarily been used therapeutically in psychiatry for people experiencing depression that has proved difficult to treat, commonly referred to as treatment-resistant depression (TRD).^2,3^ Reviews of single intravenous doses of ketamine in unipolar depression indicate that it can reduce depressive symptoms compared with either inert or active placebo, but the effects are transitory.^4,5^

Individuals with TRD often experience suicidal thinking and have increased risk of suicidal acts.^6^ The use of ketamine to try and reduce these phenomena has also received recent attention.^7–9^ Reviews of trials have demonstrated that a single intravenous treatment with ketamine can have beneficial effects on suicidal ideation, but again with transitory benefits,^10,11^ possibly of shorter duration than the effects on depression. There is no evidence regarding actual suicidal acts.^10^

Most earlier trials of ketamine treatment for depression or suicidal ideation in the context of TRD involved single infusions.^4,5,10^ Because of the often temporary benefits of a single dose of ketamine, in clinical practice multiple doses may be given and patients might be transferred to other modes of ketamine treatment (e.g. oral, intramuscular) once they have shown improvement with intravenous treatment.^12^ Research into repeated treatments has been increasing. For example, Murrough et al studied longer-term outcomes of 24 individuals following a course of six infusions over 2 weeks, and found variability in duration of positive response from 4 to 83 days.^13^ Phillips et al found that patients with a positive response in mood ratings to a single ketamine infusion, which was maximal at 7 days post-infusion, subsequently had sustained reduction in suicidal ideation with repeated weekly infusions.^14^ Review of clinical case records has indicated benefits of repeated ketamine infusions in a sizeable proportion of patients.^15^ However, there is a shortage of qualitative studies of patients’ experiences over time. Thus, little is known about patients’ views of ketamine treatment for depression, including their expectations, concerns and general thoughts about the impact of treatment in both the short and longer term. This is particularly important given that some authors have urged caution regarding provision of ketamine treatment getting ahead of evidence of effectiveness.^16,17^ Concerns have also been expressed about the impact on patients where the treatment fails, an initial impact reverses or when patients have to cease therapy because of insufficient funds to cover the cost.^18^

## The current study

The aim of this study was to explore the experiences and perspectives of patients with TRD who received ketamine treatment. We wanted to investigate their expectations of ketamine, short- and longer-term effects of treatment on mood and suicidal ideation, experiences of side-effects and overall views on ketamine treatment.

This research was carried out as part of a wider qualitative inquiry, which also involved investigating the impact of ketamine treatment on suicidal ideation.^19^

## Method

The study participants were self-funding patients attending a single ketamine clinic in the UK. Inclusion criteria were aged 18 years or over, having TRD, assessed to be an appropriate candidate for ketamine treatment and agreeable to treatment, no previous ketamine treatment for depression, capacity to consent and fluency in the English language. The criteria used by the clinic for suitability for treatment with ketamine were as follows: currently suffering from depression, have tried at least two different types of antidepressants for at least 6 weeks each at an adequate treatment dose, and have tried at least one type of psychological treatment.

Standard practice at this ketamine clinic is three intravenous ketamine infusions (0.5 mg/kg), each 1 week apart, followed by a break of 3–4 weeks, after which a clinical review takes place. At this review it is established whether patients have responded positively to treatment, i.e. treatment has resulted in a reduction of depressive symptoms to the degree that ongoing treatment is viable. At the time of recruitment into this study, further individualised treatment could take the form of regular oral ketamine (first administered at the clinic and subsequently taken at home, generally twice a week, with variable doses according to each individual's response), a combination of regular oral and intermittent intravenous ketamine, or intermittent intravenous ketamine only. No additional treatments are prescribed at the ketamine clinic, although patients typically remained on other oral antidepressants.

### Recruitment

Individuals newly referred to the ketamine clinic who were assessed to be appropriate for ketamine treatment during the recruitment phase of the project were invited to take part in the study and given participant information sheets by ketamine clinic clinicians (R.M., H.T.). Interested individuals were referred to the researcher (K.L.), who provided more information and carried out the informed consent process. Written informed consent was obtained from all participants.

Of 38 patients initially approached by the clinic staff, 12 (31.6%) agreed to participate in the study.

### Data collection

The interviews took place between May and December 2017. Participants were invited to be interviewed on three separate occasions: before treatment started, around 2 weeks after initiation of treatment and after approximately 2 months of treatment (or following completion of treatment if participants stopped receiving ketamine within 2 months). Because of the limitations of participant availability, the timings between interviews varied somewhat. Participants were also invited to keep a diary to record their treatment experiences, either in paper diary form or via the ‘notes’ section of a daily mood-monitoring platform, through which the Quick Inventory of Depressive Symptomatology (Self-Rated)^20^ was routinely completed (‘True Colours’).

One-to-one semi-structured interviews were carried out by the researcher (K.L.). Eleven out of twelve of the first interviews were face to face at the hospital site where the ketamine clinic is based, as were subsequent interviews for seven participants. The remaining interviews were via phone, Skype or Facetime.

Before each interview participants completed the Beck Depression Inventory (BDI),^21^ a widely used, 21-item self-rating scale to measure severity of depression, with each item having four possible responses scored between 0 and 3.

The first interview focused on participants’ clinical history, how they found out about ketamine treatment and their hopes, expectations and anxieties about treatment. Subsequent interviews addressed participants’ experiences of treatment, including impacts on mood and suicidal ideation, and side-effects of treatment. At the third interview participants were asked about their overall perspectives on the treatment. A copy of the interview schedules is provided in the Supplementary Appendix 1 available at https://doi.org/10.1192/bjo.2020.132.

The interviews lasted for 20–60 min and were tape-recorded for later verbatim transcribing (by K.L. and F.B.). Participants were given the opportunity to receive copies of their transcripts and, 18 months following their final interview, were invited to provide a retrospective paragraph via email with any additional reflections on treatment. Two participants requested their interview transcript and retrospective paragraphs were received from two further participants. Paper diaries were completed by three participants, and one participant kept regular notes on the electronic self-monitoring system used by the ketamine clinic (True Colours). Because of the limited response to diary-keeping and email, the results of this study are predominantly based on the interview data.

### Data analysis

The interviews were transcribed and thematic analysis carried out to identify participants’ experiences and their explanations for these, using an inductive semantic approach, following the stages of analysis recommended by Braun and Clarke.^22^ Themes were first identified in relation to individuals over the course of their interviews and then across the whole sample, using the principle of ‘following the thread’^23^ to bring the different data components together. Final agreement on themes was based on consensus discussion between two researchers (K.L. and F.B.), with supervision from L.M. The analysis was supported by NVIVO software (QSR International (2008) NVivo Qualitative Data Analysis Software (version 11); see http://www.qsrinternational.com).

### Patient and public involvement

A former patient of the ketamine clinic provided feedback on the interview schedule and participant information materials before submission for ethical approval.

### Ethical approval

The authors assert that all procedures contributing to this work comply with the ethical standards of the relevant national and institutional committees on human experimentation and with the Helsinki Declaration of 1975, as revised in 2008. All procedures involving human subjects/patients were approved by the South Central Oxford Research Ethics Committee and the Health Research Authority (reference number 17/SC/0106). The completed Consolidated Criteria for Reporting Qualitative Research Checklist can be accessed in Supplementary Appendix 2 available at https://doi.org/10.1192/bjo.2020.132.

## Results

The study sample comprised 12 individuals (6 females and 6 males), with a median age of 57 years (range 21–70 years). Participants had suffered with depression for between 10 and 50 years and two participants had additional diagnoses (bipolar disorder type 1 and obsessive–compulsive disorder). All had tried several pharmacological and psychological treatments, and three participants had received electroconvulsive therapy. Eight individuals were taking antidepressant medication at the time of recruitment into the study, with four participants taking two or more different types. Of these, two participants were also taking mood stabilisers and two participants were taking anxiolytics (one participant was taking all three types of medication). Antidepressant medication included a range of selective serotonin reuptake inhibitors, a serotonin–noradrenaline reuptake inhibitor (venlafaxine), mirtazapine, quetiapine and bupropion. Four participants were currently engaged in psychological treatments, namely counselling/therapy (*n* = 2), cognitive–behavioural therapy (*n* = 1) and psychosocial intervention (*n* = 1).

Suicidal ideation had been experienced at some point by all participants, to varying degrees of frequency and intensity, from a single fleeting thought without intent to strong ideation with reported intent and suicidal acts. Six participants had a history of self-harm. Key participant characteristics are presented in Table 1.

**Table 1:** Participant characteristics

Characteristics	*N* = 12
Gender (male/female)	6/6
Age, years	21–70; median 57
Beck Depression Inventory scores	Range 22–49; mean 32.9 (s.d. 8.5)
Past suicidal ideation	12
Suicidal ideation reported at interview 1 (Beck Depression Inventory question 9)	11
Past self-harm	6

Eight participants attended all three research interviews. Three participants attended only the first two interviews as treatment was terminated after a single dose because of side-effects (*n* = 1), or self-withdrew from treatment after two infusions because of nil or limited effect (*n* = 2). A fourth attended the first and third interviews, but had been uncontactable at the time the second interview was due.

The time points at which interviews took place varied because of availability for interview or delayed response to contact. Seven of the eleven second interviews took place at the planned time period of 2 weeks into treatment, and the remaining four took place between 1 week and 2 months after the first interview. Eight of the nine third interviews were carried out between 5 and 12 weeks after the second interview and, in the case of the participant who attended interviews one and three only, the third interview took place 4 months after the first.

Ten participants had at least three intravenous ketamine treatments, with six going on to oral treatment. Six participants were still engaged in ketamine treatment by the end of the study period. A summary of treatments received is provided in Table 2.

**Table 2:** Summary of treatments received (*N* = 12)

Mode of treatment (*n*)	Explanation for cessation or maintenance of treatment
One infusion only (*n* = 1)	Stopped because of side-effects but experienced some transient benefit
Two infusions only (*n* = 1)	Limited improvement not considered enough to justify expense and travel
Three infusions only (*n* = 2)	One non-responder; one responder with significant and sustained improvement
Four infusions only (*n* = 2)	One non-responder; one responder still engaged in intravenous treatment at end of study with significant but transitory improvement, somewhat diminished by study end
Two infusions and oral for approximately 11 weeks (*n* = 1)	Ceased treatment because of limited and diminished improvement
Infusion and oral and still engaged in treatment by end of study (*n* = 5)	One significant and sustained improvement; two ongoing improvement still present but somewhat diminished by end of study; two significant but transitory benefit, no longer present by study end

Self-reported findings from the interviews are summarised and presented below, in both numeric and narrative format, covering pre-treatment experiences and expectations, short- and longer-term impact of ketamine on mood and suicidal ideation, side-effects of treatment and participants’ perspectives on ketamine treatment overall.

### Pre-treatment: finding out about ketamine treatment and expectations of it

Most participants found out about ketamine treatment by internet searching for treatments for depression (*n* = 5) or through national news coverage (*n* = 4). Two participants were informed about the treatment by their psychiatrist and one participant noticed an improvement in mood following recreational ketamine use, which prompted internet searching for therapeutic treatment.

All participants described some sense of anticipatory hope that the ketamine treatment would stimulate changes in their mood and functioning:

‘Hopefully it might just give me the kick start to be able to get me off some medication so that I can start building up my life again.’ (P1, interview 1)

Half of the participants (*n* = 6) moderated their anticipatory hope based on past experiences of treatments not working. When asked to contemplate how they might feel if the treatment did not work, five participants said they would be disappointed but at the same time would be pleased they had tried it, four were unsure and three implied that they would feel a sense of hopelessness:

‘I don't know really. Don't know where to go from there really. Don't know what else there is. I'm just hoping it does (work). I get the impression there isn't anything else really then. I've tried most things.’ (P4, interview 1)

The main concerns about treatment were to do with it not working (*n* = 5) and side-effects, particularly the dissociative and psychotomimetic effects (*n* = 6). Two participants stated that they had no concerns at all. No participants expressed anxieties about possible development of dependency on ketamine.

### Impact of treatment on mood and suicidal ideation

Ten participants reported experiencing a positive impact on mood at some point during their course of ketamine treatment, including one who had a single infusion only before being withdrawn from treatment because of side-effects. The remaining two individuals were classed as non-responders, i.e. benefit was not enough to indicate that treatment was effective. Intensity and duration of improvements varied considerably from treatment to treatment and person to person, lasting from a few hours after treatments to some weeks. Symptomatic benefits associated with improved mood were most commonly reduced anxiety (*n* = 10), improved sense of self-worth (*n* = 9) and increased energy (*n* = 8). Three participants reported an improvement in appetite, and in three other participants this manifested as more moderated eating because of reduced urges to overeat.

The main themes relating to the lifestyle outcomes of improved mood were improved functionality (*n* = 9), becoming less self-critical (*n* = 9) and being able to socialise (*n* = 9):

‘I think the best thing is the lack of mental pain but what feels best is [physically] clearing a room……. That's kind of what gives me the (big sigh) feeling.’ (P3, interview 3)

‘I played golf yesterday and I didn't play very well but I didn't feel anxious, then we went to the pub afterwards ……. and yeah, I felt really quite relaxed if you like … I wasn't sitting there worrying about myself and worrying about what other people think of me …..’ (P4, interview 2)

#### Initial impact (first two treatments)

Nine participants noticed some improvement in mood after the first ketamine treatment: two during the infusion, four the following day and three a few days later. These improvements lasted between a day and right through to the next treatment (1 week). Generally, early effects on mood were mild (*n* = 6), although three participants described more significant benefits such as the feeling of being back to their old self (*n* = 1), feeling on form (*n* = 1) and a reduction in psychosomatic gastrointestinal symptoms (*n* = 1).

The second ketamine infusion stood out for five participants as being significantly more effective than the first, with four participants describing striking changes that were life-changing while they lasted and not experienced to the same degree following subsequent treatments:

‘I felt alive. I wasn't watching my life going past me anymore, I was part of it.’ (P2, interview 2)

‘I'd almost say it cured, it's cured, if you like, my depression…. cure, or relief from.’ (P5, interview 2)

Eight participants reported a reduction in suicidal ideation. Participants tended to associate this with improved mood. Some were starkly aware that they no longer had suicidal ideas (*n* = 4), whereas for others there was a more subtle realisation that they had not been having such ideas, or they were less intense (*n* = 4). Again, the second ketamine infusion resulted in the most profound reduction in suicidal thoughts:

‘It was like a weight had been lifted, it really was. It was quite amazing really. I could do things again; I could go out……I wasn't thinking about suicide…’ (P4, interview 2)

‘In the past [2 days] I don't think that's come into my head in anyway whatsoever. It's like, huh, why would I do that? Was I thinking about that before? Yes, I have, I've been very suicidal, now that place in my brain isn't active.’ (P3, interview 2)

Where positive effects on mood were experienced following the second treatment, these generally lasted until the next treatment, with some participants noticing a tapering of effect. This resulted in a return of suicidal ideation in some, but not all participants.

#### Subsequent impact (third treatment onward)

Ten participants had three or more ketamine treatments. Of these, two reported sustained improvements, two were non-responders and six reported that the positive effects of ketamine waned somewhat over time. In three of these six participants, suicidal ideation remained absent or reduced despite periods where improvements in mood were not sustained, but the other three participants described a return of strong suicidal thoughts as the positive effects on mood wore off. These thoughts involved consideration of suicide methods and, in two participants, some ambivalent preparatory behaviour.

Six participants progressed to oral ketamine treatment, with four noting it to be less effective than the infusions. For some, this resulted in a sense of hopelessness, whereas others maintained hope that the ketamine treatment might work again:

‘I suppose when I went to the oral after the change in the doses 2–3 times I started to lose a bit of confidence or hope in the treatment as such, that saddened me because I did think at the time I might really have a way forward but unfortunately those feelings have now diminished [……….] Initially [the doctor] described my situation as heading towards oblivion and, to be honest I feel that I'm probably going down that step again.’ (P5, interview 3)

‘It all went downhill……quite far. I can't remember whether I was actually suicidal at all, possibly not but I was very anxious for something to be fixed, to be helped…… I'm still holding onto the hope that maybe we can improve……. I'm now feeling that maybe with regular oral doses it might improve a little bit, so I'm holding onto that hope.’ (P3, interview 3)

Two individuals found oral ketamine to be more beneficial than the intravenous infusions. This was explained as possibly being a result of the cumulative effect for one participant, and because the dissociative treatment effects were less intense than with the infusions for the other participant.

Half of the participants (*n* = 6) were still in treatment at the end of the study. One of these participants described a sustained and significant improvement in mood, which had resulted in holistic positive effects:

‘I can function like I used to before I got depressed. It's given me my life back.’ (P1, interview 3)

Three of the remaining five participants reported ongoing but dwindling positive effects on mood, and two indicated that noticeable improvements had ceased. As with previous interviews, for some the fact that ketamine had worked for a time instilled hope for the future:

‘At the moment it's bad again …… there is hope now, so before there wasn't…….what I know now is that there is a chance to get better.’ (P2, interview 3)

Of the six participants who had stopped ketamine by the end of the study, one had sustained improvement in mood with a single course of three ketamine infusions and the remaining five had stopped because of non-response (*n* = 2), insufficient efficacy (*n* = 2) or side-effects (*n* = 1). One of these described feeling worse as a result of the ketamine treatment not working, and reported a sense of associated hopelessness:

‘So generally I think I feel worse…. I feel more hopeless…… Maybe I built up too much hope for it because I tried lots of other things beforehand and it seemed like this was going to be, kind of a salvation. I guess that as its not, that's probably contributed to my mood. I feel pretty kind of, oh shite, what do I do now, you know?’ (P6, interview 2)

Responses to treatment are summarised in brief in Table 2. Changes in BDI scores over the period of the study reflected participants’ narratives. These scores are shown graphically and statistically in Supplementary Appendix 2 available at https://doi.org/10.1192/bjo.2020.132.

### Side-effects of ketamine treatment

Side-effects during treatment administration were prominent, particularly dissociation, which was experienced by 11 out of 12 participants. Other effects noted were seeing or hearing unusual things (*n* = 6), blurred vision (*n* = 5) and nausea (*n* = 3). For most participants (*n* = 10) these effects lasted less than an hour or between 1 and 3 hours.

Experiences of the first intravenous ketamine treatment ranged from pleasant (*n* = 3) to unpleasant (*n* = 2), with most (*n* = 7) somewhere in between. Descriptions related mainly to dissociative and psychotomimetic effects of the treatment and wooziness or fuzzy headedness, although there was also some reference to feeling heavy (*n* = 4):

‘I felt like I was in a spaceship looking out down on the world….I felt relaxed….it was quite a pleasant experience….it was almost out of body in terms of you're almost looking at yourself as a spectator rather than actually being part.’ (P5, interview 2)

‘It was horrible. I felt very drunk and very heavy and I must have taken quite a long time, to come round…….and I think I was pretty washed out at the end of it. It was not a pleasant experience at all.’ (P9, interview 2)

Eleven participants experienced side-effects following ketamine treatment, which were reported as significant in two participants and minimal or minor in the remaining nine participants. Tiredness for a day or so after intravenous treatments was common (*n* = 6), as were headaches (*n* = 4), which for three participants were minor and continued for up to a day after treatment, although one individual experienced a debilitating 4-day long headache, which commenced 2 days after the first ketamine infusion. This was classed as a severe side-effect resulting in termination of treatment. Less common side-effects were gastrointestinal symptoms with oral ketamine (*n* = 1), and sustained sleep disturbance (*n* = 1).

Most participants did not have any concerns about ketamine tolerance or dependency, although three reflected that a sense of addiction might be a possibility:

‘I don't mind taking it because I know it's not a horrible sensation that you're getting. So I suppose in that sense you've got a slight addiction in the sense that you don't mind taking it.’ (P1, interview 3)

‘I think if it did work I probably could get addicted to it to be honest but based on them two infusions, never.’ (P11, interview 2)

One participant had some concerns about misuse of ketamine if they resorted to accessing street ketamine to avoid the cost of ongoing therapeutic ketamine treatment.

### Overall perspectives on treatment

At the last point of contact in the study, significant and ongoing improvement in mood was reported by two study participants, and a further three reported more limited residual benefits. For five other individuals, improvements had ceased and the remaining two individuals did not derive any benefit from ketamine treatment.

Ketamine treatment met or exceeded expectations for five participants. This included three participants for whom improvement had plateaued or diminished. Three individuals were clear that their expectations had not been attained. Others said they had not held any expectations (*n* = 2) or that they had been met in part (*n* = 2).

The expense of treatment was problematic for some participants (*n* = 5), especially when they were not deriving ongoing benefit:

‘I feel quite dispirited really. Because it's been really expensive. You know, I haven't been anywhere or done anything because all my money has gone on this, so I don't feel great about it at the moment.’ (P7, interview 3)

The short-lived effect of treatment was frustrating for participants who experienced transient benefit, from a cost perspective and from resulting difficult emotions and fluctuating levels of hope:

‘My treatments with ketamine were wholly beneficial for myself and greatly improved my mood although only for a relatively short time. If the cost of the treatment was reduced, I would like to have had more treatments but at the present time I lack the necessary funds.’ (P4, email correspondence)

‘I was a bit angry and sad…….. I couldn't believe it [improved mood] stopped and [I was] just back the way I was. It's sad because, I hope if in the future I'm going to do this again, I kind of want to do it and hopefully this is going to get better with longer treatment, but if it doesn't it, you know, it makes me think that, what's the point of living.’ (P2, interview 3)

Participants who did not derive lasting benefit from ketamine were nevertheless generally positive about the availability of an alternative to traditional antidepressants. Some volunteered helpful suggestions for improving treatment experiences, such as more individualised dosages (*n* = 2); closer monitoring by clinic staff via phone calls or email (*n* = 3); candid discussion of the need to manage expectations, including patients being made aware of that there were no guarantees of benefits, the risk of relapse and possible short duration of effects (*n* = 2); and to balance the financial implications with an uncertain outcome (*n* = 3). One participant was keen that adjunctive psychological therapy should be offered alongside ketamine treatment, and others reported being able to make better use of existing psychological therapy (*n* = 2) or openness to engaging in therapy (*n* = 2).

## Discussion

In this qualitative study we investigated the experiences and perspectives of patients undergoing ketamine treatment for TRD. As far as we are aware, this study is the first to examine patient experiences over time. Most participants involved in this research derived some benefit from ketamine, but there was considerable variability in terms of intensity and duration, ranging from negligible and/or short term to substantial and sustained improvement. The main benefits described were improved mood and reduced suicidal ideation, with associated positive outcomes of improved functionality and socialisation and reduced anxiety and self-critical thoughts. However, loss of effectiveness over time and consequent declines in hope were marked in some. For those who derived benefit, improvements were maintained in two participants, diminished to varying degrees over time in six participants and waned quickly and completely in two participants.

Our findings reflect those from case report studies of a similar number of individuals with TRD who were tried on maintenance ketamine therapy,^24,25^ which also showed that most patients experienced initial but not ongoing improvement.

### Impact on mood

Noticeable improvements in mood occurred at varying time points and for differing lengths of time, ranging from days to weeks. This reflects what is known from trials that suggest improvement in mood usually lasts for between 1 and 2 weeks, longer in a minority of cases.^4,5,8^ In our research the second intravenous ketamine treatment resulted in marked improvement for some participants, which lasted longer than benefits following the other ketamine treatments. Some patients went on to oral treatment with ketamine following the initial intravenous treatment, but with mixed responses. It is recognised that bioavailability of ketamine delivered by the oral route is relatively lower.^26^

### Impact on suicidal ideation

Most participants who reported a reduction in suicidal ideation associated it with improvement in mood. For some, however, suicidal thoughts remained alleviated even when their mood began to deteriorate. This indicates a possible independent effect, which has been tentatively identified in recent reviews of the impact of ketamine on suicidal ideation^10,11^ and in a previous qualitative study.^19^

### Side-effects and other consequences of ketamine treatment

Reflecting wider research into the effects of ketamine treatment,^13,27,28^ participants in this study perceived the main side-effects of ketamine treatment to be those experienced during intravenous treatment administration, particularly dissociation and strange or unreal sensations, which were transient and resolved shortly after treatment. Side-effects following treatment were mainly fatigue and headaches, which are again commonly reported in the literature.^28^

A minority of our participants thought that there might be a possibility of dependency on ketamine occurring over time. Dependency has not been identified as an adverse outcome of ketamine treatment,^27^ although concerns about misuse potential have been raised,^18,29-31^ including risk of illegal procurement. One individual in our study referred to a possibility of purchasing street ketamine to self-medicate because it was cheaper and more locally accessible than therapeutic ketamine treatment. This view has also been expressed by actual or potential ketamine patients in other studies.^18,32^ The possibility of this occurring cannot be ruled out for some patients, particularly given the widespread availability and low cost of recreational ketamine.

Most participants in our study found out about ketamine via the media, suggesting that widespread reporting attracts individuals who are searching for novel treatments to provide respite from depression. A sense of desperation for effective treatment has been reported by prospective ketamine patients,^32^ and concerns have been expressed that such desperation might lead individuals to home in on the reported benefits of ketamine and overlook the limitations, leading to dashed hopes.^31^ Similar experiences have been reported for other treatments that are sometimes used in severe mood disorders, such as deep brain stimulation, where media hype and the challenges of trying to modify unrealistic expectations are common features.^33,34^ Hope was a large part of the ketamine treatment experience: hope that it might work, hope when it did work, hopelessness (in some cases) when it stopped working and hope that it might work again. For some participants, hopelessness was related to the impossibility of long-term treatment because of lack of funds, which suggests that these feelings might be related both to beneficial effects of ketamine wearing off and concerns about availability of alternative treatments. Similar cases are described elsewhere,^17,35^ which have contributed to suggestions that adjunctive psychosocial interventions^17^ and closer monitoring of ketamine patients^35^ may augment beneficial outcomes and ensure early identification of relapse. These suggestions have also been made by patients.^18^ Some individuals in our study identified a need for closer monitoring and, with regard to psychological input, one participant thought that it should be provided soon after treatment-related dissociative and psychotomimetic effects have worn off because, in their experience, receptiveness will be optimised at this point because of improved mood and openness to the possibility of change.

### Strengths and limitations

A strength of this study is that it was conducted in a routine clinic setting rather than a trial, so there were no formal inclusion/exclusion criteria beyond that participants had TRD and were treated with ketamine. Although the results of this study are not necessarily generalisable, the qualitative approach adopted is advantageous in that it provides a nuanced understanding of experiences of patients and thus a more complete overview of the effects of ketamine treatment than clinical trials alone. Given the heterogeneity of patients with TRD, clinical trials that adopt pragmatic inclusion criteria may attract a wider diversity of patients, including those without financial means to pay for treatment. We suggest that such trials should incorporate nested qualitative studies to enable patient experience to be included to provide more complete study outcomes.

The small number of participants in this study is a limitation, as is the relatively short duration of follow up. Also, because of participant availability, there was some inconsistency in the time periods between interviews and the number of interviews attended, which, along with the variations in treatment courses based on the individualised approach of the clinic, may have added to the variability of responses. However, these factors represent normal aspects of routine provision of treatment with ketamine. Self-report from the semi-structured interviews rather than validated clinical scales was largely relied upon when seeking information about changes/improvements to mood and hopelessness, to gain a full understanding of patients’ experiences. However, the BDI was used to obtain a more objective measure of mood at key points throughout the study. All participants had the financial means to fund treatment, which does not reflect the wider TRD population, most of whom would not be able to afford self-funded treatment.

Recently, delivery of ketamine in the form of esketamine, the S-enantiomer of ketamine, via a nasal spray has been developed, with some promising results when combined with an antidepressant.^36^ Although the USA Food and Drug Administration has approved the use of esketamine in combination with an antidepressant for TRD^37^ and the European Commission has given similar approval,^38^ the UK National Institute for Health Care Excellence has not approved its use in the National Health Service.^39^ We do not know if the findings of this study would be reflected in patients receiving esketamine nasal spray treatment according to the licensed doses.

In conclusion, the findings from this qualitative study of patient experiences when receiving treatment with ketamine complement those from treatment trials. They indicate some benefits for mood and suicidal ideation in the majority of patients, but with reductions in these benefits over time for most, but not all, initial responders to treatment. They also suggest that there should be caution regarding overly optimistic interpretation of the place of ketamine treatment in the management of TRD. Informed consent procedures should include reference to the high likelihood of relapse. Together with the current cost of treatment, especially for individuals who cease to be able to afford it, this has the potential to increase hopelessness. There is also a major need to investigate whether adjunctive therapies, especially psychological ones, can help sustain early benefits of treatment.

## Data Availability

Because of the qualitative nature of this study, full transcripts of the interviews cannot be made available as these could potentially identify study participants.

## Supplementary material

For supplementary material accompanying this paper visit http://dx.doi.org/10.1192/bjo.2020.132. click here to view supplementary material

## References

[1] Duman RS. Ketamine and rapid-acting antidepressants: a new era in the battle against depression and suicide. F1000Res 2018; 7(F1000 Faculty Rev): 659 10.12688/f1000research.14344.1 PMC5968361 29899972

[2] Gaynes BN, Lux L, Gartlehner G, Asher G, Forman-Hoffman V, Green J, Defining treatment-resistant depression. Depress Anxiety 2020; 37: 134–45. 31638723 10.1002/da.22968

[3] McAllister-Williams RH, Arango C, Blier P, Demyttenaere K, Falkai P, Gorwood P, The identification, assessment and management of difficult-to-treat depression: an international consensus statement. J Affect Disord 2020; 267: 264–82. 32217227 10.1016/j.jad.2020.02.023

[4] McCloud TL, Caddy C, Jochim J, Rendell JM, Diamond PR, Shuttleworth C, Ketamine and other glutamate receptor modulators for depression in adults. Cochrane Database Syst Rev 2015; 9: CD011612. 10.1002/14651858.CD011612.pub2 26395901

[5] Corriger A, Pickering G. Ketamine and depression: a narrative review. Drug Des Devel Ther 2019; 13: 3051–67. 10.2147/DDDT.S221437 PMC6717708 31695324

[6] Oquendo MA, Currier D, Mann JJ. Prospective studies of suicidal behavior in major depressive and bipolar disorders: what is the evidence for predictive risk factors? Acta Psychiatr Scand 2006; 114(3): 151–8. 16889585 10.1111/j.1600-0447.2006.00829.x

[7] Price RB, Iosifescu DV, Murrough JW, Chang LC, Al Jurdi RK, Iqbal SZ, Effects of ketamine on explicit and implicit suicidal cognition: a randomized controlled trial in treatment-resistant depression. Depress Anxiety 2014; 31(4): 335–43. 24668760 10.1002/da.22253 PMC4112410

[8] Grunebaum MF, Galfalvy HC, Choo TH, Keilp JG, Moitra VK, Parris MS, Ketamine for rapid reduction of suicidal thoughts in major depression: a midazolam-controlled randomized clinical trial. Am J Psychiatry 2018; 175: 327–35. 29202655 10.1176/appi.ajp.2017.17060647 PMC5880701

[9] Ionescu DF, Bentley KH, Eikermann M, Taylor N, Johnson-Akeju O, Swee MB, Repeat-dose ketamine augmentation for treatment-resistant depression with chronic suicidal ideation: a randomized, double blind, placebo controlled trial. J Affect Disord 2019; 243: 516–24. 30286416 10.1016/j.jad.2018.09.037

[10] Witt K, Potts J, Hubers A, Grunebaum MF, Murrough JW, Loo C, Ketamine for suicidal ideation in adults with psychiatric disorders: a systematic review and meta-analysis of treatment trials. Aust N Z J Psychiatry 2020; 54: 29–45. 31729893 10.1177/0004867419883341

[11] Wilkinson ST, Ballard ED, Bloch MH, Mathew SJ, Murrough JW, Feder A, The effect of a single dose of intravenous ketamine on suicidal ideation: a systematic review and individual participant data meta-analysis. Am J Psychiatry 2018; 175: 150–8. 28969441 10.1176/appi.ajp.2017.17040472 PMC5794524

[12] Andrade C. Ketamine for depression, 4: In what dose, at what rate, by what route, for how long, and at what frequency? J Clin Psychiatry 2017; 78: e852–7. 28749092 10.4088/JCP.17f11738

[13] Murrough JW, Iosifescu DV, Chang LC, Al Jurdi RK, Green CE, Perez AM, Antidepressant efficacy of ketamine in treatment-resistant major depression: a two-site randomized controlled trial. Am J Psychiatry 2013; 170: 1134–42. 23982301 10.1176/appi.ajp.2013.13030392 PMC3992936

[14] Phillips JL, Norris S, Talbot J, Hatchard T, Ortiz A, Birmingham M, Single and repeated ketamine infusions for reduction of suicidal ideation in treatment-resistant depression. Neuropsychopharmacology 2020; 45: 606–12. 31759333 10.1038/s41386-019-0570-x PMC7021716

[15] Sakurai H, Jain F, Foster S, Pedrelli P, Mischoulon D, Fava M, Long-term outcome in outpatients with depression treated with acute and maintenance intravenous ketamine: a retrospective chart review. J Affect Disord 2020; 276: 660–6. 32871698 10.1016/j.jad.2020.07.089

[16] Schatzberg AF. A word to the wise about ketamine. Am J Psychiatry 2014; 171: 262–4. 24585328 10.1176/appi.ajp.2014.13101434

[17] Talbot J, Phillips JL, Blier P. Ketamine for chronic depression: two cautionary tales. J Psychiatry Neurosci 2019; 44: 384–5. 31573153 10.1503/jpn.190073 PMC6821514

[18] Jilka S, Murray C, Wieczorek A, Griffiths H, Wykes T, McShane R. Exploring patients’ and carers’ views about the clinical use of ketamine to inform policy and practical decisions: mixed-methods study. BJPsych Open 2019; 30: e62. 10.1192/bjo.2019.52 PMC6669880 31530293

[19] Lascelles K, Marzano L, Brand F, Trueman H, McShane R, Hawton K. Effects of ketamine treatment on suicidal ideation: a qualitative study of patients’ accounts following treatment for depression in a UK ketamine clinic. BMJ Open 2019; 9: e029108. 10.1136/bmjopen-2019-029108 PMC6701814 31420388

[20] Rush AJ, Trivedi MH, Ibrahim HM, Carmody TJ, Arnow B, Klein DN. The 16-Item Quick Inventory of Depressive Symptomatology (QIDS), clinician rating (QIDS-C), and self-report (QIDS-SR): a psychometric evaluation in patients with chronic major depression. Biol Psychiatry 2003; 54: 573–83. 12946886 10.1016/s0006-3223(02)01866-8

[21] Beck AT, Steer RA, Carbin MG. Psychometric properties of the Beck Depression Inventory: twenty-five years of evaluation. Clin Psychol Rev 1988; 8: 77–100.

[22] Braun V, Clarke V. Using thematic analysis in psychology. Qual Res Psychol 2006; 3: 77–101.

[23] Moran-Ellis J, Alexander VD, Cronin A, Dickinson M, Fielding J, Sleney J, Triangulation and integration: processes, claims and implications. Qual Res 2006; 6(1): 45–59.

[24] Archer S, Chrenek C, Swainson J. Maintenance ketamine therapy for treatment-resistant depression. J Clin Psychopharmacol 2018; 38: 380–4. 29912788 10.1097/JCP.0000000000000894

[25] Chan LF, Eu CL, Soh SY, Maniam T, Kadir ZS, Chong BTW, Is ketamine the future clozapine for depression? A case series and literature review on maintenance ketamine in treatment-resistant depression with suicidal behavior. J Psychiatr Pract 2018; 24: 279–91. 30427812 10.1097/PRA.0000000000000316

[26] Harrington R, Whittaker J, Shoebridge P, Campbell F. Systematic review of efficacy of cognitive behaviour therapies in childhood and adolescent depressive disorder. BMJ 1998; 316(7144): 1559–63. 9596592 10.1136/bmj.316.7144.1559 PMC28555

[27] Wan LB, Levitch CF, Perez AM, Brallier JW, Iosifescu DV, Chang LC, Ketamine safety and tolerability in clinical trials for treatment-resistant depression. J Clin Psychiatry 2015; 76: 247–52. 25271445 10.4088/JCP.13m08852

[28] Short B, Fong J, Galvez V, Shelker W, Loo CK. Side-effects associated with ketamine use in depression: a systematic review. Lancet Psychiatry 2018; 5: 65–78. 28757132 10.1016/S2215-0366(17)30272-9

[29] Bobo WV, Voort JLV, Croarkin PE, Leung JG, Tye SJ, Frye MA. Ketamine for treatment-resistant unipolar and bipolar major depression: a critical review and implications for clinical practice. Depress Anxiety 2016; 33: 698–710. 27062450 10.1002/da.22505

[30] Schwartz J, Murrough JW, Iosifescu DV. Ketamine for treatment-resistant depression: recent developments and clinical applications. Evid Based Ment Health 2016; 19: 35–8. 27053196 10.1136/eb-2016-102355 PMC10699412

[31] Singh I, Morgan C, Curran V, Nutt D, Schlag A, McShane R. Ketamine treatment for depression: opportunities for clinical innovation and ethical foresight. Lancet Psychiatry 2017; 5: 419–26. 10.1016/S2215-0366(17)30102-5 28395988

[32] Veraart JKE, Smith-Apeldoorn SY, Trueman H, de Boer MK, Schoevers RA, McShane R. Characteristics of patients expressing an interest in ketamine treatment: results of an online survey. BJPsych Open 2018; 4: 389–92. 30202601 10.1192/bjo.2018.51 PMC6127959

[33] Schlaepfer TE, Lisanby SH, Pallanti S. Separating hope from hype: some ethical implications of the development of deep brain stimulation in psychiatric research and treatment. CNS Spectr 2010; 15: 285–7. 20448518 10.1017/s1092852900027504

[34] Bell E, Maxwell B, McAndrews MP, Sadikot A, Racine E. Hope and patients’ expectations in deep brain stimulation: healthcare providers’ perspectives and approaches. J Clin Ethics 2010; 21: 112–24. 20866017

[35] Schak KM, Voort JLV, Johnson EK, Kung S, Leung JG, Rasmussen KG, Potential risks of poorly monitored ketamine use in depression treatment. Am J Psychiatry 2016; 173: 215–8. 26926127 10.1176/appi.ajp.2015.15081082

[36] Zheng W, Bin CD, Xiang YQ, Jiang WL, Sim K, Ungvari GS, Adjunctive intranasal esketamine for major depressive disorder: a systematic review of randomized double-blind controlled-placebo studies. J Affect Disord 2020; 265: 63–70. 31957693 10.1016/j.jad.2020.01.002

[37] US Food and Drug Administration (FDA). FDA Approves New Nasal Spray Medication for Treatment-Resistant Depression; Available Only at a Certified Doctor's Office or Clinic. FDA, 2019 (https://www.fda.gov/news-events/press-announcements/fda-approves-new-nasal-spray-medication-treatment-resistant-depression-available-only-certified).

[38] European Medicines Agency. Summary of Opinion (Initial Authorisation), Spravato, esketamine. European Medicines Agency, 2019 (https://www.ema.europa.eu/en/documents/smop-initial/chmp-summary-positive-opinion-spravato_en.pdf).

[39] National Institute for Health Care Excellence (NICE). Nasal Spray Medicine for Treatment-Resistant Depression not Recommended by NICE. NICE, 2020 (https://www.nice.org.uk/news/article/nasal-spray-medicine-for-treatment-resistant-depression-not-recommended-by-nice). 40587641

